# Patient Satisfaction with Pre-Hospital Emergency Services. A Qualitative Study Comparing Professionals’ and Patients’ Views

**DOI:** 10.3390/ijerph15020233

**Published:** 2018-01-30

**Authors:** Fernando García-Alfranca, Anna Puig, Carles Galup, Hortensia Aguado, Ismael Cerdá, Mercedes Guilabert, Virtudes Pérez-Jover, Irene Carrillo, José Joaquín Mira

**Affiliations:** 1 Departament de Salut, Sistema d’Emergències Mèdiques, 08908 L’Hospitalet de Llobregat, Spain; fernandogarcia@gencat.cat (F.G.-A.); apuigmari@gencat.cat (A.P.); carlesgalup@gencat.cat (C.G.); 2 Servei Català de la Salut, CatSalut, 08028 Barcelona, Spain; haguadob@gmail.com (H.A.); icerda@catsalut.cat (I.C.); 3 Departamento de Psicología de la Salud, Universidad Miguel Hernández, 03002 Elche, Spain; mguilabert@umh.es (M.G.); v.perez@umh.es (V.P.-J.); icarrillo@umh.es (I.C.); 4 Centro de Salud Hospital-Plá, Departamento de Salud Alicante-Sant Joan, 03550 Alicante, Spain

**Keywords:** pre-hospital emergency, review, qualitative study, patient satisfaction

## Abstract

*Objective*: To describe patient satisfaction with pre-hospital emergency knowledge and determine if patients and professionals share a common vision on the satisfaction predictors. *Methods:* A qualitative study was conducted in two phases. First, a systematic review following the PRISMA protocol was carried out searching publications between January 2000 and July 2016 in Medline, Scopus, and Cochrane. Second, three focus groups involving professionals (advisers and healthcare providers) and a total of 79 semi-structured interviews involving patients were conducted to obtain information about what dimensions of care were a priority for patients. *Results:* Thirty-three relevant studies were identified, with a majority conducted in Europe using questionnaires. They pointed out a very high level of satisfaction of callers and patients. Delay with the assistance and the ability for resolution of the case are the elements that overlap in fostering satisfaction. The published studies reviewed with satisfaction neither the overall care process nor related the measurement of the real time in responding to an emergency. The patients and professionals concurred in their assessments about the most relevant elements for patient satisfaction, although safety was not a predictive factor for patients. Response capacity and perceived capacity for resolving the situation were crucial factors for satisfaction. *Conclusions:* Published studies have assessed similar dimensions of satisfaction and have shown high patient satisfaction. Expanded services resolving a wide number of issues that can concern citizens are also positively assessed. Delays and resolution capacity are crucial for satisfaction. Furthermore, despite the fact that few explanations may be given due to a lack of face-to-face attention, finding the patient’s location, taking into account the caller’s emotional needs, and maintaining phone contact until the emergency services arrive are high predictors of satisfaction.

## 1. Introduction

In the 1950s, Koos [1] proposed that patients be listened to in terms of what they had to say about the healthcare they received. Shortly thereafter, Donabedian [2] laid the foundations for the current conception of quality in the healthcare sector by definitively incorporating the patient’s perspective as a measure for the healthcare outcome. Then, some years later, Doll [3] asserted that healthcare must be evaluated by considering clinical effectiveness, efficiency, and acceptance by the patient for the care provided.

The concept of satisfaction has been related to attitudinal aspects, wherein the components had a distinct value depending upon the patient’s personal situation, and it was conceptualized as the result of the difference between how the patient had been attended to and his expectations about what such care should have been like [4]. Until well into the 20th century, the instruments for evaluating patient satisfaction had developed in an environment wherein the health system was centered on the professionals and not the patients. The changes promoting patient-centered care have led to the search for alternative methodologies.

Starting in the 1970s, patient satisfaction measures spread throughout the health services [4,5], commonly including evaluation of the following dimensions [6,7]: accessibility, professional competency, aspects of comfort and the physical appearance of facilities, availability of equipment, empathy of the professionals, information (quantity and quality) provided by the professionals, possibilities for choice, response capabilities of the professionals, and continuity of care between distinct care levels. Practically all research has focused on patient satisfaction following their discharge from the hospital or primary care. 

The number of studies published regarding the satisfaction of patients who have accessed pre-hospital emergency services is limited considering the large numbers of patients who annually utilize this service (7,147,754 healthcare demands in 2015 in Spain [8]). Although some similarities might occur, there are expected differences in cases of an emergency. Moreover, in recent years, some countries that use an emergency telephone number have introduced new benefits such as completing administrative procedures, providing health advice over the phone that leads to a solution, instructing the user to go to a health center, and dispatching mobile units or health professionals to the location whether it is a public place or the caller’s home. In these cases, patients also speak to a highly trained adviser, supported by healthcare professionals, and the motive for calling is different from an emergency.

The aim of this study was to describe the knowledge about patient satisfaction with pre-hospital emergencies (telephone support, care provided, and emergency healthcare transport) and determine if patients and professionals share a common vision on the key variables for satisfaction.

## 2. Materials and Methods

This is a qualitative study conducted in two phases. First, a systematic review of the literature was carried out to identify methods applied to assess patient satisfaction with pre-hospital emergencies via the use of emergency telephone numbers (e.g., 911 in the USA and 112 in Europe) to request medical assistance or information from pre-hospital emergency services and search for improvement opportunities from their results. Second, three focus groups and a total of 79 semi-structured interviews were conducted to obtain information about what dimensions of care were a priority for patients and professionals (pre-hospital providers: physicians, nurses, and dispatchers). Dispatchers were trained to offer support over the telephone during an emergency. They use algorithms to make decisions and provide information or instructions. In this study, a pre-hospital emergency was defined as a demand for providing care for out-of-hospital health urgencies and emergencies. This includes telephone support, care provided in situ, and emergency healthcare transport. Pre-hospital emergencies are demanded by callers. Such callers are patients who dial the emergency telephone number (112 or 061) while in some cases, these calls are placed by others (relatives, friends, or anybody else) who help the patient by calling, asking for support.

### 2.1. First Phase: Literature Review

The PRISMA protocol was followed to analyze published research, both in English and Spanish, on satisfaction of the patient (of any age) with emergency telephone assistance, requests for emergency assistance, and emergency transport to a hospital. Publications (both quantitative and qualitative research) indexed between January 2000 and July 2016 in Medline, Scopus, and Cochrane were reviewed by combining the following MeSH terms: emergency telephone number, emergency telephone call center, out-of-hospital emergency telephone, pre-hospital emergency telephone, caller satisfaction emergency, medical services, out-of-hospital emergency, pre-hospital emergency, and ambulance, which were combined with the Boolean operator along with caller satisfaction and patient satisfaction.

The following inclusion criteria were established: research on any type of population, from elderly patients to parents of pediatric patients. Differences were not made based on the reasons for the call, pathology being dealt with, or who was making the call (the caller). All types of calls were included, no matter whether they were local, national, or international.

Excluded research included studies dealing with hospital emergencies, those that only described or evaluated the clinical attention during some part of the process (from the moment the call was placed until care was begun by the mobile unit, for example) without assessing the satisfaction of the attended user, those that described coordination mechanisms between units, those that assessed the satisfaction of professionals, and those that analyzed the quality of the decisions that healthcare professionals had to make (redirect the call or not, send a vehicle or something else, perform the intervention within the transport, or refer to the hospital).

The studies were reviewed independently by two of the authors to decide whether they fulfilled the inclusion criteria using the titles, abstracts, or full article. The final decision was made jointly by both of them. Additional articles were retrieved from the reference lists of the articles found by the initial online search. From the articles selected, the following information was categorized: year, country, objective, method or measurement, sample, evaluated dimensions of perceived quality, and outcomes.

### 2.2. Second Phase: Qualitative Research

This study, based on a qualitative research approach (using focus groups and semi-structured interviews), evaluated and compared the perspective of professionals with that of patients about what elements of perceived quality are most relevant for users of the services of the Sistema d’Emergències Mèdiques (Emergency Medical System, named SEM in Spanish) of Catalonia.

The Emergency Medical System is a public entity, dependent upon the Servei Català de la Salut (Catalonian Health Service), and is responsible for attending to, managing, and responding to demands for providing care for out-of-hospital health urgencies and emergencies in Catalonia. It serves 7 million people in an area of 32,000 km^2^. Its main access is via the telephone, with patients dialing 112 for emergencies and 061 for other health demands. The operational structure for the provision of this service includes a coordination center, 406 mobile units (326 basic life support and 80 advanced life support ambulances), and 4 medical helicopters.

Its coordination center is charged with taking and managing telephone calls. In 2016, it dealt with 1,473,609 cases, which was a 6% increase over 2015. Of these cases, 40.4% (595,156) were resolved without activating care resources, achieved by the consulting efforts and information provided by the professionals there. In patient care by pre-hospital emergency systems, two areas can be clearly differentiated: distance care and on site care. Distance care is provided by the professionals at the call reception center. The range of activities carried out at this level varies depending upon the central model. In the specific case of the SEM, these would be the following: taking the call, locating the patient, and triaging the situation with a computerized protocol. These functions are taken care of by the dispatchers, and based on the outcome of the initial triage, the call may be transferred to a second level of telephone dispatcher when the caller needs administrative information of a certain complexity; the call may also be transferred to health professionals, physicians, or nurses. This latter group asks about the patient’s medical history over the phone in accordance with some clinical procedures to better define the patient’s needs. The call may then finalize with the provision of health advice, or care in situ may be deemed necessary, resulting in the mobilization of a resource (ambulance or medical helicopter). When mobilizing a resource is deemed necessary, the coordination center decides upon the most suitable type depending upon the isochrone map and the patient’s pathology, it coordinates the activated resources, and when necessary, the transport to the health center, deciding which is most suitable depending upon its distance and saturation level, information that the coordination center has available at all times. On site care is that provided by the professionals from the teams of the care resources, ambulances, and helicopters. Once they reach the patient, care consists in learning the patient’s medical history, exploration, treatment and, if necessary, transport to a health center with care provided during the ambulance ride.

Telephone calls by citizens are handled by two types of professionals: first there are demand managers (dispatchers), and then there are pre-emergency providers, and these include physicians and nurses. Dispatchers are responsible for taking calls, and after a short consultation with the caller and interaction using a computerized protocol, a response may be generated or the call may be referred to a second level of attention. These professionals have experience and have been trained on providing care for patients via telephone support platforms, and prior to working professionally at the SEM, they receive specific training on the tools to use, certain skills for telephone assistance in urgencies and emergencies, operational SEM protocols, and also on basic health knowledge. They also undergo continuous training. In the latter case, and provided the demand is for informative content, the call is attended to by a group of dispatchers who are not health professionals but nonetheless can spend greater time responding to the citizen in an appropriate manner. These types of calls make up the group of administrative consultations. Issues related to the services provided by the Catalonian Health Service are the main reason for telephone consultations, and in 2016, there were 211,702 such administrative consultations. In the event that a call referred to the second level corresponds to a consultation on a health urgency, the call is transferred to either a physician or a nurse (depending upon the content of the consultation). In 2016, there were 324,821 of these health consultations.

**Figure 1:**
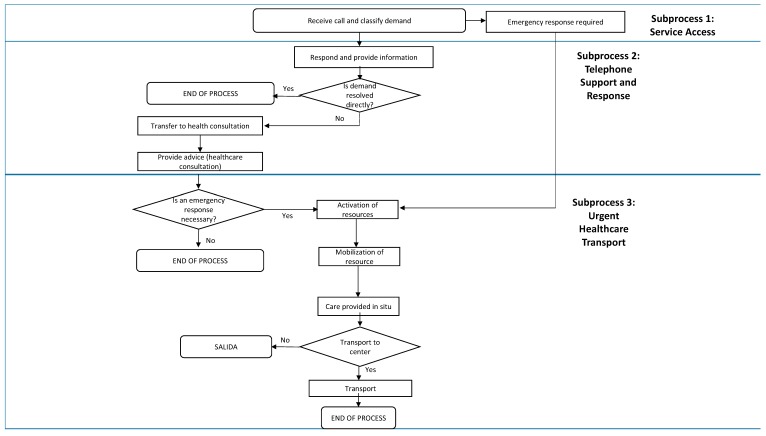
SEM-061 care process.

Figure 1 details the analyzed SEM-061 care process that was assessed, divided into three subprocesses. Subprocess 1: Service Access (accessibility to the care line). Subprocess 2: Telephone Support and Response (call answered and classified, assessment of the assistance provided over the phone). Subprocess 3: Emergency Healthcare Transport (mobilization of care resource, arrival of care resource and care provided in situ, decision about transport, care provided during transport, and lastly, the transition to the health center). This study explored the different subprocesses that began when the patient (or somebody else) dialed 112 or 061 and until they either received care at the location they were found in or were transported to a healthcare center, and finally whether the patient was transferred to the hospital, and until the emergency care process was considered finished.

### 2.3. Perception of Professionals

Three focus groups were led by 23 professionals (14 pre-hospital providers from the coordination centers of Reus and Hospitalet (pre-hospital emergency 061 CatSalut Respon) and 9 professionals who provided care at the second level, urgent health transport). Sixteen semi-structured interviews (see Supplementary Table S1) were conducted with telephone dispatchers from the pre-hospital emergency 061 CatSalut Respon service. Participation was voluntary after the study objectives and methodology were explained to them. The selection of these professionals considered their professional experience (less than 6 months, 1–3 years, or more than 3 years) on different shifts (morning, afternoon, evening, weekend), and there was equal representation from areas (urban and rural) in addition to balance between men and women.

### 2.4. Perception of Patients

To select users who called SEM, a random sample of 264 callers who had used both the 112 and 061 services was identified from the CatSalut billing database. Of these users, 119 (45%) called for administrative or health consultations, and the remaining 145 (55%) requested urgent medical transport and were conscious when the care arrived in order to be able to report on the care they had received. Prior to conducting the semi-structured interview, each caller was asked whether they retained sufficient memory to assess different aspects of the quality of the service they had received. As such, 34 (13%) said that they did not retain sufficient memory of the events to report on the care that they had received, 41 (16%) were very active users of both emergency and non-emergency transport services but unable to assess the quality of both services differently, and 66 (25%) declined to participate in the interview.

Ultimately, 63 callers (24%) participated in semi-structured interviews lasting 10–15 min; 33 of them had called for administrative consultations whereas the remaining 30 had dialed 061 for healthcare requests (see Supplementary Table S2). Additionally, and in the case of emergency medical transport, a subsample of 55 callers (22%) for emergency medical transport from different areas of Catalonia were interviewed in depth to attain an approximation of the user population for this service.

The inclusion criteria included the following: be between 18 and 90 years of age and a caller of 061 in the preceding 6 months (September 2016–February 2017) for either an administrative or a health consultation. Those requesting emergency medical transport had to have called during the preceding year (2016) for the following possible varieties: advanced vital support (SVA), basic vital support (SVB), medical helicopter, rapid intervention vehicle (VIR), or the unit of continuous home care. Likewise, geographic origin was taken into account for callers of administrative and health consultations alike, as well as for callers requesting urgent health transport, since the territorial variable was a variable that could affect perception; thus, an attempt was made to distribute the sample in rural and urban areas.

## 3. Results

### 3.1. First Phase: Literature Review

**Figure 2:**
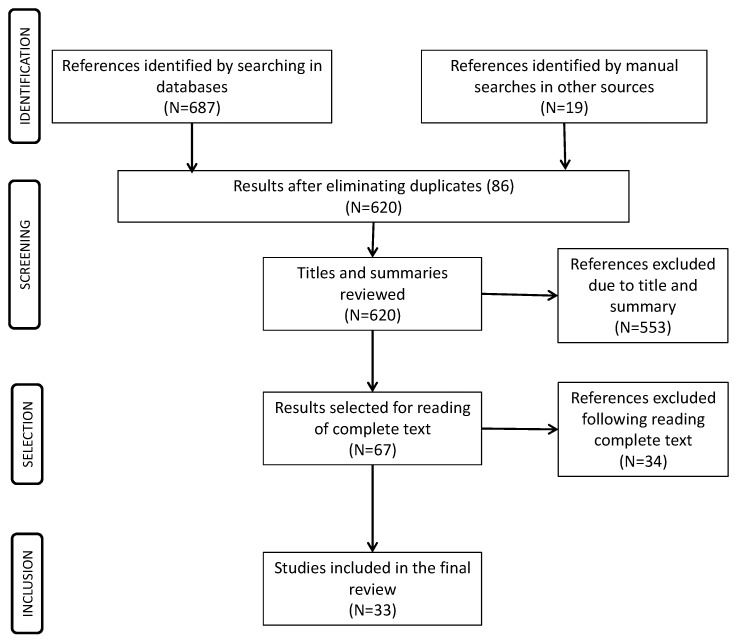
PRISMA figure. Results of the literature review.

The search strategy produced 620 additional studies that were of potential interest for this study, and after reviewing their titles and abstracts, this figure was reduced to 67 (Figure 2). After completely reading those 67 texts, 33 relevant studies were identified (Table 1).

Most of the studies published on satisfaction with the telephone assistance provided by pre-hospital emergency services are of the descriptive variety (71%) that employed either surveys, questionnaires, or structured interviews with patients. Most of these were carried out in the United Kingdom (41%). Of the reviewed studies, 24% of them were systematic reviews of the literature, although not always using the PRISMA methodology. None of the published studies reviewed the overall care process; instead, they focused on parts of the process such as telephone triage, communications skills of the attending professionals, if the recommendations were ultimately carried out, and whether there was any follow-up.

The most frequent origin for published studies on emergency healthcare assistance that included transport, normally via ambulance (none inquired about other transport means, such as helicopter, water craft, etc.), was once again the United Kingdom (36%). The majority of these were descriptive studies (75%) that coincide in pointing out a very high level of satisfaction of callers and patients, although the fact of being attended to by various telephone dispatchers, technicians, or healthcare professionals during the same phone call is indicated in these as practically the lone cause for dissatisfaction. Delay with the assistance and the ability for resolution of the case are the elements that overlap in fostering satisfaction. No studies relate the measurement of the real time in responding to an emergency with the satisfaction of patients.

The care elements that are recognized as generating confidence and professionalism include short wait times, receiving information on the reasons for the transport, the patient’s expectations coinciding with the action taken by the professional (for example, being transported or not), and attending to both the physical as well as emotional aspects of the assistance. Studies that address the problems of language difficulties have not been found, for example, assistance for foreigners in tourist areas or cultural differences due to religion that require differentiated treatment, such as male/female relationships.

Entirely all the studies on satisfaction with emergency services show that in all countries, continents, and systems, such as Malaysia, Japan, USA, Europe, and Australia, patients report feeling very satisfied with the dimensions evaluated in the instruments employed (treatment perceived as adequate, information, delay, conditions of transport, capacity for resolution) [9,10,11,12,13,14,15]. However, these approaches have not provided information for identifying improvement opportunities, for example on patient safety. Sharing the vision of professionals and patients could probably be a good complement to identify opportunities for care process improvement. Analyzing the care elements that influence more directly on this satisfaction and exploring alternative methodological approaches to opinion surveys could provide qualitatively different information for learning about the experiences of patients who require these services. Published studies have focused on patients using ambulances, but no patients were included who required air or maritime transport.

### 3.2. Second Phase: Qualitative Research

The patients (callers) and professionals (dispatchers and pre-hospital providers) concurred in their assessments about the most relevant elements for the patient when patients use the emergency telephone and also in the assessments they both made about said service (Table 2 and Table 3). Likewise, they also agreed on the quality criteria for patients when mobilizing a unit such as an ambulance or helicopter is required (Table 4).

#### 3.2.1. Accessibility

Patients generally know what telephone number to call in emergencies or when they need health information, and they use this telephone service correctly. In the same manner, both groups (patients and professionals) agreed that the new communication channels (chat, email, apps) were not sufficiently known about or utilized, even for patients who usually accessed, due to their domestic situation, the emergency telephone number. However, the time required to resolve the call were higher when patients used 112 because this is a general entrance for all type of emergencies including health emergencies. Patients calling 112 usually needed to repeat the same information to a second professional.

#### 3.2.2. Response Capacity

Both groups pointed out the delay by which telephone assistance is carried out as a critical criterion of quality, and the patients stated this was adequate. The professionals, for their part, properly sensed that the delay in providing care was key, and that the assessment by patients in this regard was positive. The main gap of having to repeat practically the very same information again and again when the phone call is transferred to different professionals during the same call was identified by both groups; one example of this is when the call is transferred after the patient has activated her medical alert button and already talked first to the providers of that service. Furthermore, in these cases, there could be sporadic delays or even waits before the physician or nurse returns the call a few minutes later (adjusted according to urgency).

The professionals emphasized that if the caller was not the patient, obtaining reliable information was more complicated, and this aspect was also recognized by patients during the interviews.

As for mobilizing resources (ambulance, helicopter, etc.), the impression conveyed by patients is that a delay in receiving assistance is the factor they valued most when judging the efforts by the emergency service and that, in all cases, the resource arrived quickly. The professionals agreed on that assessment, although they pointed out difficulties when the caller did not know about his location, or the problems of finding him in certain rural locations, especially mountainous terrain. Moreover, one variable that professionals pointed out that leads to delays in attending to the emergency call is when the caller is a tourist and does know exactly where he is; this makes pinpointing his exact location difficult.

#### 3.2.3. Professionalism

Another relevant aspect for patients is the professionalism in how the situation is handled, both when gathering information from the patient as well as when offering structured information in a logical and comprehensible manner. Patients assessed both of these aspects positively. Furthermore, the professionals correctly sensed the assessments that patients made. In general, the comments by patients who were interviewed indicate that they felt that they were correctly understood, including emotional aspects that accompany uncertainties during emergencies.

Although patients did not indicate to be a problem the fact that telephone operators did not remain on the line with them once the emergency assistance request was made until the resource arrived at their location, the professionals did agree in pointing out that in this care, also attending to the emotional needs of patients entails a higher level of quality. Patients and professionals alike agreed in their assessments that the resources mobilized brought the appropriate means and professionals for dealing with the emergency care request and that, therefore, a suitable use of the means available was made. Patients and their companions (when applicable) received correct information about the patient’s situation, the treatment being administered, and where they were being taken.

#### 3.2.4. Transport Conditions

Patients did not pay special attention to the safety conditions during the emergency transport whereas professionals did value them and considered them important. However, the patients’ opinions about these conditions were that the transport was carried out correctly and safely. Callers of pediatric patients described how the transport went about for these minors (a child of theirs) in a manner that was consistent with the care protocol that the professionals described during the interviews for this young population. When the parents were aware of this protocol during the interview, they assessed it positively.

#### 3.2.5. Capacity for Resolving the Situation

Both groups agreed on this aspect as crucial for determining the quality of the care and in the positive assessments made by patients in this regard. There were many emergencies that were resolved in the same place as the incident (home, hotel, beach, street). Thus, the capacity for resolving emergencies was not only related to safety and fast transportation to a hospital.

The patients stated that the transport took place quickly and without incident, their personal belongings were not lost, and that in the transition to the emergency hospital personnel attending to them, teams there took charge of their care in the terms in which they had been informed. The professionals pointed out this transition as a critical point for patient safety and that the information between professionals and the patient should improve. The professionals also pointed out that a protocol should be applied to ensure that personal belongings are returned to patients.

## 4. Discussion

Treatment perceived as adequate, information about diagnosis, treatment and hospital to be transferred to, delays, conditions of transport, and capacity for resolution are the dimensions of patient satisfaction usually explored in the literature. More than 85% of patients are usually satisfied with pre-hospital emergency services, whereas repeating the same information when being served by several operators is a cause for dissatisfaction.

The dimensions of satisfaction identified in this study coincide with those indicated in the literature as keys to the satisfaction of these patients. The results of this study have yielded new aspects to be considered, such as professionalism also including that the telephone operator is capable of finding the location from which the call requesting assistance is placed when the caller himself does not know where he is, and that the professionals also attend to the emotional needs of the person making the call.

Patients, dispatchers, and healthcare providers share the crucial assistance and care quality perceived dimensions. The professionals correctly identify what patients consider crucial when receiving information or care and their level of satisfaction. These results also coincide that satisfaction with these services is very high. The expectancies of patients about emergencies are related to delays and resolution capacity while professionals considered other aspects related with safety, a dimension not considered by patients.

Emergency services have expanded their services and offer agile and reliable information on a wide number of issues that can concern citizens and, for that matter, patients, such as resolving doubts about the correct use of a medication, information about how to correctly interpret a medical indication, knowing where to go when in search of health assistance, and requesting emergency assistance at home. This change has been shown to be useful and that it achieves a positive impact on the user population [27]. The results of this study confirm these initial assessments, although they indicate that when defining the emergency care processes, they should consider that the organization should prevent the caller from repeating the same information to various telephone operators.

From a methodological point of view, these results show that while the memory of the ambulance ride (if the clinical conditions so allow) remains over time, the same does not happen with memory when dialing 061 to obtain timely information, even during stressful moments. The reality is that, as was shown previously [9], users of this service learn quickly to raise doubts over the phone, and their assessments when asked are overall ones because most of them do not remember the last time that they called. Also, when these services are assessed, the diversity and complexity of the health service being offered must be kept in mind. For example, it is necessary to take into account that when conducting evaluations of these health services, a large number of emergencies are resolved in the location where they take place and do not require transport in an ambulance or any other mode of transportation.

The result of studies on patient satisfaction have highlighted some of the predictors of satisfaction such as [42,43,44,45] age, intimacy, and cleanliness, length of hospital stays, knowing what type of professional they were dealing with at any moment, information at admission and about home care after discharge, patient-reported experiences with the nursing and physician services, perceived the treatment as correct, and fulfillment of patient expectations. In the case of the emergency services, this study revealed as variables important to satisfaction that the caller perceives that their necessities are understood by the telephone operator and that telephone contact is maintained during the time they wait for the ambulance to arrive.

There is a concentration of studies in Europe. The generalization of results to other countries might be limited as this uses a qualitative research approach. There was no random selection of professionals involved in focus groups. The assessments were not related with objective measures of assistance such as length of phone assistance, delays, claims or adverse events.

As far as we have been able to determine, this is the first study that examines the entire care process that is carried out by emergency service providers, and it is designed to learn about what care aspects are relevant for patients and whether the professionals keep these dimensions in mind. Its qualitative methodology allows an alternative approach to that of survey-based studies and, as seen in this case, provides information for introducing improvements in the care process.

## 5. Conclusions

Worldwide, there is high satisfaction with pre-emergency hospital services. The expanded services offering agile and reliable information on a wide number of issues that can concern citizens also yield high satisfaction. Pre-emergency patients, dispatchers, and healthcare providers share the crucial assistance and care quality perceived dimensions. 

The dimensions of satisfaction usually assessed include delays and resolution capacity that are crucial for fulfilling patients’ and callers’ expectation about this service. Finding the location from which the call requesting assistance is placed, maintaining contact with the patient until the emergency services arrive, and attending to the emotional needs of the person making the call are high predictors of satisfaction. These are key elements when callers (and patients) assess the professionalism of the dispatchers and the healthcare providers. Repeating the same information when being served by several operators is the principal cause for dissatisfaction.

When satisfaction is assessed, the role of callers and patients must be considered because many times the callers cannot provide reliable information.

This study describes some methodological issues to be considered when satisfaction instruments are developed and the gaps and strengths of the pre-emergency care that could contribute to improve the satisfaction of callers (and patients).

## Figures and Tables

**Table 1:** Description of studies included in final review.

Yes	Country	Objective	Measurement Method	*N*	Dimensions Assessed of the Perceived Quality	Most Relevant Results
2015 [16]	Austria and Switzerland	Evaluate patient satisfaction regarding the call, treatment, transport, and hospital admission into the emergency medical service in Austria and Switzerland.	Survey, multicentric study	Austria: 291 (response rate, 44.5%) Switzerland: 240 (response rate, 49.7%) Total: 531 (response rate, 46.7%)	4 dimensions: emergency call, emergency treatment, transport, and hospital admission. 48 quality indicators: wait time, time dedicated to patient, treatment, skills of healthcare personnel (medical, emotional, listening, social, education, friendliness), cordiality, professionalism, treatment efficacy (pain relief), information, decision making, culture, intimacy, comfort during transport, safety during transport.	In 91.7% of cases, the general satisfaction with the emergency treatment obtained a very high score (between 90 and 100 points). The average scores in all quality criteria evaluated exceeded 90 points (out of 100) except for some items referring to the social skills of healthcare professionals (85.8 medical, 84.8 emotional, 86.3 listening, 83.3 social).
2015 [17]	United Kingdom	Assessment of the quality and safety of emergency care.	Systematic review	45 reviews and 102 empirical studies	--	Satisfaction with telephone triage varied between 55–97%. Dissatisfaction between 2.3–18.3% was greater when patients expected to be supplied with an ambulance. There was less satisfaction with nurses than with physicians.
>2015 [18]	>United Kingdom	Review of studies on ambulance use by primary care patients.	Systematic review	Of 31 studies, 1 study satisfaction	--	Satisfaction decreases sequentially depending upon the number of different services that establish contact with the patient before receiving definitive care.
2015 [19]	Saudi Arabia	Degree of knowledge of the emergency service in Jeddah.	Survey in public places	1534 residents of the city of Jeddah	Knowledge of the number to call, ambulance request, time for ambulance to arrive, preference for a single centralized telephone number or one for each service, confidence of the paramedics in the treatment performed, coverage, trust, whether a male may attend to a female patient without a male relative present.	22% considered ambulance coverage adequate. 32% waited less than 30 min for the ambulance. 18% considered that if no male was within the residence, nobody could enter it to provide care for a female.
2014 [20]	United Kingdom	Aspects that ambulance users value.	Interview: personal (*n* = 18) and telephone (*n* = 12)	22 patients and 8 wives (*n* = 30)	Positive and negative aspects of the experience with the ambulance services.	Peace of mind was emphasized. Peace of mind that they were receiving adequate advice, treatment, and care by the attending ambulance personnel. The professional behavior by the personnel provided peace of mind, the confidence they show in the care, communication, a short wait for receiving assistance, and continuity during transfers.
2013 [9]	Denmark	Learning the perception that patients have about the entire “chain of survival” before arriving at the hospital, from the moment they dial 112 until they arrive at the hospital, as well as the impact that using different levels of urgency has on the patient’s overall impression.	Mail survey	1419 (response rate, 58%)	General impression, care, treatment, information, confidence, response time.	In general, 98% of patients who called 112 defined the pre-hospital care as either “very good” (82%) or “good” (16%). The overall impression was more positive in cases when patients had been informed about the expected response time and when the evaluation of the degree of urgency by the SEM coincided with the patient’s self-evaluation. The level of urgency perceived by the patients was often lower than that evaluated by the SEM. The highest scores were observed in relation to how the ambulance was driven (91%), the confidence in how the ambulance personnel (including pre-hospital nurses and physicians) handled the situation (91%), and the respect by the personnel (90%). The lowest scores were related to the apparent preparation of hospital personnel upon patient arrival (72%), sufficiently rapid ambulance arrival (74%), and sufficient involvement by family members (77%). The specific aspects that had a high relationship with the patient’s overall impression and obtained low scores were the following: care provided by calling 112 [79.1 (75.8–82.4)], ability of the ambulance personnel to explain to them what was being done to them [79.3 (77.1–81.6)], and involvement by family members in accordance to the needs of the patient [76.9 (74.0–79.7)].
2013 [21]	Sweden	Describe the experiences of elderly patients who have used pre-hospital emergency services after a fall due to a suspected hip fracture.	Interview in patient’s homes	10 elderly patients	Experience with the ambulance service.	3 themes emerge: efficiency (the service was efficient, structured, and rational); that relative to the meeting (in the relationship that is established, during the meeting, between the personnel and patient the elements highlighted most are the dialog and tact, as well as the combination of empathy and medical knowledge); and suffering (they feel they are excluded from the decisions and confused by the drugs to relieve pain).
2013 [22]	Germany	Investigate the effects of the sociodemographic factors of the patient or his/her relatives with the satisfaction with the care of the pre-hospital emergency services.	Paper and online questionnaire	57 immigrant users and 161 nonimmigrant users	First part: sociodemographic characteristics (age, gender, education level, birthplace of patients, and their nationality). Second part: factors related to incidents occurring during the emergency services (sensations, emotions during the care provided by the emergency service). The third part analyzed factors related with the service and the experience with the emergency service. Its last section analyzed the overall satisfaction with the pre-hospital emergency service.	Most aspects related to satisfaction are explained by aspects related to the service rather than by sociodemographic aspects. The fact of being an immigrant does not show a significant relationship whereas the fact of having better or worse German language skills was related negatively with satisfaction. Professional, social, and emotional competencies were related significantly with the satisfaction of hospital pre-emergency services. (See annex: questions asked)
2012 [23]	Australia	Analyze what factors of patients were determinant in the paramedics for not taking them to the hospital.	Semi-structured telephone interview	20 patients	Experiences of the participants in the adopted decision, the factors that influenced such decision, and the consequences of not being transported to the hospital.	The reasons for not going to the hospital were varied (they only sought assistance, advice and/or support; the problem was resolved before the ambulance arrived; they did not want to go to the hospital for personal reasons, etc.). On most occasions, they did not remember the advice given to them by the paramedics or whether they had advised them about anything. All patients expressed high satisfaction with the ambulance service.
2012 [10]	New Zealand	Determine the experience and opinions of patients in relation to the first extended care paramedic (ECP) before considering its implementation in other locales within the region. Determine if patients consider that the Urgent Community Assistance model is effective and acceptable, and whether there are differences in satisfaction with the care provided by groups of paramedics: standard service of urgent ambulance and extended assistance.	Face to face or telephone survey (depending upon the patient preference)	100 (50 patients who had been attended to by the standard urgent ambulance service and 50 attended to by urgent community assistance)	Wait times (from placing the call until the ambulance arrives; from hospital arrival until care begins); time of dedication; treatment; satisfaction with evaluation at home; clarity of the information about treatment; preference on assistance location (home vs. hospital); information about what to do in case of deterioration; general satisfaction with care received; information about transport to hospital; comfort during transport (ambulance).	Patient satisfaction with the care received was very high regardless of the group of paramedics that cared for them, and the location wherein they were cared for (9.5–9.6/10 home; 9.8/10 hospital). 100% considered that the treatment received by paramedics was adequate. 77.8% preferred or would have preferred to be cared for in their home. 93.5% of patients who received healthcare in their home considered that they had been informed clearly about the treatment. 91.8% of patients who were transported felt comfortable in the ambulance and 96.2% received information on the reasons for the transport.
2012 [24]	USA	Determine if a relationship exists between the perception of the quality of the care and satisfaction of patients who have used healthcare transport regarding treatment for pain experienced in pre-hospital settings.	Retrospective review of patient satisfaction data	2741 patients	Patient satisfaction scale using a 5-point Likert scale (Excellent to Poor). Scale items: knowledge and abilities of EMS personnel, attentive and caring attitude, instructions, or explanations about the treatment or tests, explanations given about any medication and its secondary effects with respect to race and culture, and the availability of the technology necessary for treating the patient in situ. As for the quality of the care provided, they are asked with a direct item.	65.9% indicated that the care received was excellent. Of the patients who indicated that the treatment for managing pain was excellent when using non-severe transport services, 79.0% affirmed that the overall quality of the care was excellent, while only 21.0% of those patients indicated that they had received excellent overall care when the treatment for pain management was not. When the patients indicated that the emergency medical personnel was excellent in terms of the assistance given for reducing pain and explaining the treatment, they were 2.7 (95% confidence interval: 1.4–5.4) times more likely to claim that the overall quality of the care was excellent.
2012 [25]	United Kingdom	Describe experiences of patients with angina pectoris or infarction who have used ambulances.	Semi-structured interview	22 patients	Experiences with 4 main issues: communication, professionalism, treatment of the problem, and transport from home to the hospital.	For patients, knowledge and relational skills contribute to the perception of professionalism. They emphasize professional-patient communication as a key element, and the experience is more positive when they feel that their physical, emotional, and social needs have been addressed.
2011 [26]	Sweden	Evaluate patient satisfaction with the healthcare assistance received from the ambulance service by using the Consumer Emergency Care Satisfaction Scale (CECSS).	Scale of measurement of patient satisfaction with the nursing care in the emergency room	40 patients from two different regions (20 from each one)	Measures patient satisfaction with the care, competence, and education.	The average time of assistance by ambulance was 31 min. 93.1% of the participants chose the most positive response option for each question on the scale. The item valued most was “The nursing personnel took time to attend to my needs.” On this, all participants marked the most positive option.
2011 [27]	United Kingdom	Explores the acceptability of the lone telephone number for urgent care, NHS 111.	Mail survey to those who had called 111	1769 responded (41% response rate), of which 872 supplied comments (49% of those who responded)	Multidimensional satisfaction: aspects of patient-centered care (relief, assistance from personnel), access (clarity upon when to use the service), communication and information (importance of the questions posed), technical quality (whether they advised correctly), and efficiency (speed in solving their problem).	75% indicated that the advice given had been very useful, and 28% said it was sufficiently useful. Most of those surveyed (86%) indicated that they fully complied with the advice. 63% were very satisfied, and 19% were sufficiently satisfied with the service in general. Users were less satisfied with the relevance of the questions asked and with the accuracy and relevance of the advice given than with other aspects of the service. Users who were referred to call NHS 111 were less satisfied than those who had called directly.
2010 [28]	Portugal	Systematic review of evidence on the TTAS (Telephone Triage and Advice Services), the impact they have on the healthcare systems, and the methods and measures used in these studies.	Systematic review of the literature	55 papers	The studies reviewed on satisfaction use Likert-type scales to evaluate the quality of this telephone service.	In the specific case of studies of TTAS satisfaction, there are high levels of satisfaction declared by patients; however, in the studies reviewed, satisfaction is less when the TTAS constitutes a barrier to traditional care (for example, home visits).
2009 [29]	Sweden	Assess user perception of telephone helpline handled by nursing.	Qualitative research. Unstructured interviews	12 service users	Accessibility, perception about how the user was treated, and about the quality of the recommendation.	Accessible and trustworthy operators. Respect, courtesy, response capacity, correct self-care recommendations in the opinion of the users assessed positively.
2009 [30]	Spain	Describe the quality perceived by external users of a non-medical health transport unit.	Interviews were given within the hospital upon arrival or after returning home.	317 external users of the non-medical health transport of Alicante	Satisfaction of external customers with the service received: if an ambulance was requested by telephone or personally, when it was requested, the time it took the ambulance to pick them up, help and assistance to enter and exit the ambulance, driver friendliness and manners, courtesy shown to companion(s), time in transport, picking up other external customers on the same route, comfort during the trajectory, incidents during the trajectory, interior and exterior cleanliness of the ambulance, driving, and safety.	For 92.7% of users, the wait times for transport were under an hour, while in 7.2% of cases, the customer waited between 1 and 2 h. Transport was provided for services of rehabilitation and external consultations. When asked if they would recommend this service, 60.9% said they would surely recommend it, while 39.1% said they might recommend it. 57.7% of users said that the telephone service received to ask for transport was very good. The personal attention received in this service was assessed as very good by 53.3% of users. 52.6% indicated that the help and assistance during the service were very good. The friendliness and manners shown was assessed as very good by 60.2%. Courtesy and treatment shown toward companion(s) was valued as very good by 42.2%. 12.6% indicated that the comfort during the transport was very good. 29.3% thought that the ambulance interior was very clean. The driving and safety during transport were considered very good by 13.2% of its users.
2008 [31]	USA	Evaluate the effectiveness of a pediatric nursing telephone counseling program in terms of satisfaction with the service and access to care by the guardians/parents of the pediatric patients.	Quasi-experimental study with 2 samples before introducing the telephone counseling program through nursing, and then again 8 months after program implementation	Pre-sample with 14 subjects (parents and guardians of children). Post-sample with 20 subjects (parents and guardians of children)	Questionnaire to evaluate the satisfaction and results of the telephone counseling given over the telephone. 19-item questionnaire: 13 Likert-type scale items, 5 closed-answer questions, and one open question.	The results showed that the parents/guardians of the pediatric patients who formed part of the nursing telephone counseling program were more satisfied with the advice provided, according to the telephone call, and the fact of making them participate in the decision making. The year prior to the implementation of the telephone counseling program, the call reception center received 5850 phone calls, while subsequent to implementation of the telephone service, the number of calls rose to 6003.
2008 [11]	Malaysia	Obtain a measure of the satisfaction of patients with the ambulance service at the Sains Malaysia University Hospital.	Interview given before arriving at the hospital	87 patients who used the ambulance service	Vehicle (5 items), attitude (5 items), transport (5 items), professionalism (5 items), efficiency (3 items), and image (1 item).	The scores for all elements evaluated varied between 9.3 and 9.7 out of 10.
2007 [12]	United Kingdom	Evaluate satisfaction with a new care service with paramedics for elderly persons with mild illnesses.	Experimental study. Mail questionnaire taken 28 days after calling 999	3018 patients over 60 years of age (*n* = 1549 interventions, persons who had been attended by this new paramedic counseling service, and *n* = 1469 control)	General satisfaction.	Patients included in the experimental group indicated to a larger extent to be “very satisfied” than those in the control group (85.5% vs. 73.8%, *p* < 0.001).
2007 [13]	USA	Evaluate the quality of the medical emergency service (a paramedic service that establishes the type that the patient requires for transport to the hospital).	Mail survey to all those who had used the service from 2001 until 2004	851 patients who used this service	Courtesy, clarity of information, and overall satisfaction.	In the four years in which they were surveyed, 99.5% were satisfied.
2007 [32]	United Kingdom	Analyze the perception of patients attended to in the emergency services.	Questionnaire on satisfaction with the emergency services	43 users of the services	As a measure of the quality perceived in the patient questionnaire: wait time before being attended to, confidentiality and respect for rights, personalized attention, applied counseling, ability to listen and understand, delivery of informed consent.	Good performance translates into elevated patient satisfaction results.
2007 [33]	United Kingdom	Evaluate the adequacy, satisfaction, and cost of the emergency professionals (ECP, Emergency Care Practitioner). Increase the understanding of the effect, if any, that emergency professionals have in offering healthcare services on a local level. Evaluate whether ECP efforts save costs.		524 patients (245 experimental group (treated by ECP) and 279 in the control group (treated by the usual professional)	General satisfaction, future preference.	Three days after initial contact with the service, a greater number of patients from the ECP group than those from the control group affirmed to be “very satisfied” with the consultation (85.4%, *n* = 105 vs. 66.4%, *n* = 85). 77% (*n* = 100) of patients seen by the ECP said that in the future they would prefer to be treated by ECP professionals instead of any other type of healthcare professional. The emergency professionals provided more treatments (Chi^2^ = 26.0, *p* < 0.001, df = 1) and advice (Chi^2^ = 8.0, *p* < 0.001, df = 1). The ECP were more likely than the professionals from the control group to discharge patients at their very home instead of transporting them to the hospital, and of those who answered the follow-up questionnaire, it was more probable that the patients were very satisfied (Chi^2^ = 6.2, *p* < 0.001).
2006 [34]	United Kingdom	Compare the experience of patients who received care from emergency healthcare personnel (emergency care practitioners, ECP) with other patients who received care from traditional ambulance professionals (paramedics registered by the state or emergency medical technicians).	Mail questionnaire	888 (response rate, 53.6%)	General satisfaction with the care: wait time, courtesy, attitude, listening, information, relevance of the treatment, adequacy of evaluation, comfort.	For most aspects related to the care, the majority of participants assigned “very” positive scores, although the frequency of these was less than 50% of that referring to “explanations about what would happen afterwards” (35.1–44.5%), “information provided” (38.0–44.8%), and “feel comfortable with what has happened” (40.6–45.1%). It was more likely that patients attended to by emergency healthcare personal, in comparison with those cared for by personnel of traditional ambulance services, to score “very” positively the “explanation by personnel about what would happen” (OR = 1.5; 95% CI = 1.1–2.1) and the “thoroughness of the evaluation” (OR = 1.4; 95% CI = 1.0–1.9) (although the difference in the latter was not maintained when controlling the variation in the transport). In short, the experiences of patients attended to by emergency personnel and those attended to by traditional ambulance services (paramedics or technicians) were similar and generally positive. However, in two areas, the care provided by emergency personnel was considered better.
2005 [35]	Sweden	Determine how patients with acute chest pain experience the emergency call and their pre-hospital care.	Semi-structured interview (open questions)	13 patients	How was your experience?	The patients express fear in that the telephone operator would not understand the seriousness of their problem. For patients who were alone, the ambulance wait was difficult and they were scared they would lose consciousness and that nobody would find them. Contact with the telephone operator was important for the patients, and the information that the ambulance was in route reassured them. Patients who had previously had an unsatisfying experience with a telephone operator hesitated at the time of making the call and took longer to complete it. For the patients, it was important that the treatment begin at home and continue along the way to the hospital and for the telephone operator to confirm that they would be attended to (“everything is going to be fine”). The patients said they felt safe upon hearing they would receive professional help and that the ambulance was well equipped. The secure feeling increased in cases in which the patient had the opportunity to remain in contact with the patient until the ambulance arrived. The patients stressed the importance of adequate information during the call (that the ambulance is on the way and the approximate wait time). Patients’ confidence increased when they were the center of attention, the treatment was individualized, and a highly qualified professional came to their home.
2005 [36]	United Kingdom	Evaluation of the clinical effectiveness and cost-effectiveness of ECP care (a new professional figure from the emergency service who attends, filters, and diverts patient care in emergencies) in England on different telephone service routes.	Controlled observational survey. It measures satisfaction during one of the study phases with patient surveys.	524 patients who answered the survey	They measure satisfaction on a Likert scale in accordance with the following items: personnel had manners, personnel were worried about me, personnel listened to me, personnel responded to my questions, personnel performed a thorough medical examination, medical treatment was excellent, satisfaction with the recommendations and advice given, and general satisfaction with the care received.	In each item, the results were better among those who were treated by this new professional profile.
2005 [37]	United Kingdom	Assess the joint work between nurses and paramedics when attending to a medical emergency in the home, reducing the number of patients who must attend the hospital emergency services. To accomplish this, the experiences of both professionals and patients were explored.	Qualitative research: interviews and focus groups. Paper surveys sent to patients of the pilot service (paramedic and nurse) and to the user who received the standard service (only paramedics).	64 patients (27 from the pilot group and 37 from the standard group) 11 patients involved in Focus Groups	In the patient questionnaire, the main categories analyze: Reasons for calling an ambulance. Experience when the emergency service arrived and the treatment. Perception of the care received. Following the advice received. Among the group of professionals, the main categories analyzed (before and after implementing the pilot experience): Opinion about the introduction of the service. Expectations about the types of calls that the teams of nurses and paramedics must attend to. Possible benefits and drawbacks of the service. Skills and knowledge gained. Concerns. After implementation of the pilot experience: Number of calls taken, types, changes. Facilitators and inhibitors of the new service. Experience about the teamwork. Benefits and drawbacks of the new service. Skills and knowledge utilized.	Patients who received the pilot service were very enthusiastic about the opportunities of receiving care in their own home. It was also a positive experience for the professionals. They underwent greater job satisfaction and increased their skills and knowledge.
2004 [38]	United Kingdom	Develop and evaluate protocols for ambulance personnel when attending to non-serious cases, providing advice on self-care and referring to other services without using health transport.	Experimental study. With a battery of items, they measure satisfaction of the users of the service. The scale is a 5-point Likert.	251 in the experimental group and 537 in the control group	They measure satisfaction with a battery of items: The ambulance personnel listened attentively when I spoke about my problem, the people were friendly, the number of tips given by them were adequate, a sensation of calm after talking with the physicians, satisfaction with the explanations, clear advice about when I must request more help, general level of satisfaction with the service personnel, they made us feel like we were wasting the physicians’ time.	A greater portion of the patients in the experimental group declared themselves satisfied.
2003 [39]	USA	Evaluate the quality and cost of the telephone service based on a triage system by nursing.	In-person survey or a telephone survey (as per patient preference).	300 tutors of pediatric patients aged 0 to 16 years who utilized the Computerized Telephone Nurse Triage system (CTNT).	SERVQUAL Questionnaire administered via the telephone. This contains 16 items grouped into 4 dimensions on a 7-point Likert scale. This questionnaire measures quality of the services based on perceptions: Sociodemographic variables: gender, relationship with child, age, education, type of employment, number of times call center has been utilized, age of child, gender of child, date of birth, number of children in the family.	The average SERVQUAL score was 6.42. For the confidence dimension, the score was 6, it was 6.71 for the sensitivity dimension, assurance of quality scored 6.47, and the empathy dimension was 6.65. Most users who called: working mothers assessed the quality of service highly. Neither the education, level of employment, age of the user, gender of the child, year of birth, whether the children were twins, nor the age of the children affected the quality of the service when evaluating. Parents (male) of children assessed the quality of the service at a lower level. The method of telephone triage is well accepted as an alternative to providing healthcare that can be useful for many parents of children.
2003 [14]	Australia	Analyze the levels of satisfaction with ambulance services in the Australian state of Victoria.	Questionnaire	Cannot be determined		Those surveyed are more satisfied with the ambulance services than with the emergency services.
2002 [40]	United Kingdom	Analyze the acceptability of a telephone assistance system for emergencies (Emergency Medical Dispatch).	Mail surveys sent to two randomly selected samples of 500 users who had called 999 beforehand, and one year after implementing the EMD service.	355 users from before the EMD system was implemented answered the system (72% response rate), along with 297 users one year after implementation (63%)	Speed by which the telephone was answered, number of questions asked, importance of the formulated questions, returning the call when necessary, satisfaction with the telephone call, whether advice was given, advice was not given but was necessary, satisfaction with the quantity of tips, use of the tip (if given).	There was a decrease in users who considered that all questions asked were relevant (81% vs. 70%), which did not affect the percentage of users who were very satisfied with the call to 999, which moved from 78 to 86%. Satisfaction levels with the quantity of counseling increased (35% vs. 56%). The proportion of those surveyed who were very satisfied with the service in general increased from 71 to 79%. In the written comments, two problems were detected: some users were given advice to carry out actions that subsequently proved to be unnecessary; and second, a small number of persons who called felt that the ambulance crew did not treat the situation as seriously as they would have liked.
2002 [15]	USA	Analyze the effect of an educational program for ambulance medical professionals and a quality improvement cycle, which helps physicians decide upon the need for transporting patients over 65 years of age in an ambulance who have not been transported.	Pre- and post-observational study. Telephone interviews with patients over 65 years of age who had contact with the ambulance physician but were ultimately not transported.	151 patients in the first phase and 109 patients in the second phase	One direct item that measures satisfaction.	In the first phase, 94.7% declared themselves satisfied. In the second phase, 100% declared themselves satisfied (OR = 0.08; *p* = 0.03).
2000 [41]	United Kingdom	Analyze the usefulness of the new service, NHS Direct, 24 h of telephone assistance with nursing personnel, which was implemented to “provide information easier and faster for persons about health, illness, and the NHS so they become more capable of caring for themselves and their family members.”	Mail questionnaire sent to those who had called during a specific week of September 1998, which was sent a week after their call and, and subsequently two reminders.	719 answered (71%), of which 579 left written comments (81%)	Usefulness of the nursing advice, the reason why the service was useful (peace of mind, help in contacting the correct service, learning to address problems on their own, avoiding contacting a service, learning to avoid future problems, carried out the advice (yes, completely; yes, some; no).	Most answered that the nursing advice was very or sufficiently useful (643; 95%); many carried out the advice given (566; 85%). The most common reason they found the advice useful was that it gave them peace of mind (425; 66%).

**Table 2:** Subprocess 1: Service Access.

Professionals		Caller/User	
1. Accessibility			
1.1 Telephone (061/112 service)			
Confusion about the services offered by 061/112 “Some still keep referencing the former ‘*Sanitat Respon*,’ they think they are calling 112;” “People are very confused;” “People today, because of all the TV ads for 112, think that 112 is for everything. Even when the primary care centers have to provide information, they say to call 112, and the chain starts from there…”	13	Health and administrative consultations recognize 061 as a support service. 112 is mostly for emergency consultations “The last time I called, almost a year ago, I was having a heart attack. My brother was with me and I said we’re going to call because I had very strong chest pains, and they came right away.”	8
1.2 Ignorance about alternatives to dialing 061/112			
Use of other alternatives (chat/email) to a lesser extent by users			
“The main criticism of users, and why they use other channels, is the cost of the call. Users are asking you about urgent things by chat. My water just broke, I’m having contractions, or my daughter is choking, by chat…” “And emails to avoid paying for the service.”	11	“I made the consultation by email.” “I know about the mobile app, but I prefer to use the telephone.” “I didn’t know there were other ways to contact them.” “Email is slower than calling, because when calling, they respond right away.”	6
1.3 Types of consultations made			
Administrative and health consultations “Fever, consultations about fever;” “Anxiety … suicidal thoughts. ”“They would tell you about vaccinations, they would give you information related to the trip…;” “The type of health consultations is not for urgent things, but rather about fevers, vaccinations.” “Information about their health card.”	11	Greater demand for health consultations“ The telephone must be used for health emergencies.” “I called because I needed to make a consultation outside of my primary care center’s operating hours.” “It is an alternative to visiting your primary care center.”	11
1.4 User/Caller/Affected individual as persons who initiate the support service			
Greater difficulty for resolving the problem when the caller is not the affected person“ A lot of people call who are not those affected.” “Many times the wife calls us, the wife of a 40–50 year-old who is right next to her, then she begins asking him questions, and you end up asking if she can just give him the phone.”	15	The caller does not realize that the assistance can be complicated when making contact with the affected person is not possible“ It wasn’t me who called; it was a family member who did.” “The last time I called, almost a year ago, I was having a heart attack. My brother was with me and I said we’re going to call because I had very strong chest pains, and they came right away.”	3

**Table 3:** Subprocess 2. Telephone Support and Response.

Professionals		Caller/User	
2. Call answered and classified			
2.1 First initial contact with the telephone operator			
Rapid initial response if the call is made to 061 or 112			
“There are problems; a call reaches us after the patient had activated their medical alert button…” “Elderly people push the button, they talk to the medical alert people, and medical alert has neither resources nor physicians nor anything, so they transfer the call. Many times elderly callers are not aware that they are speaking with 061.”	3	“They are very fast;”“I didn’t have to wait;” “They are relatively fast.” “They answered the telephone quickly.” “I waited between 3 and 5 min.”	5
2.2 Phone transfer to health professional			
The phone transfer from the telephone operator to the specialist is perceived as the moment when a delay in care occurs			
“The user calls and the telephone operator classifies the call, and tells the user that they will call him back as soon as possible.” [When there are other emergencies]. “You have to follow an algorithm because you have to guarantee that the information reaches the channels, then the protocol, sometimes depends upon whether the operator is new … It does not stop being a superficial tool as sometimes it turns out that if it is a hemorrhage, an ambulance is sent due to protocol, but just the same, a grandfather is calling you because he’s been having a slight nose bleed for all of three minutes. Then sometimes things are activated outside of protocol … an ambulance is sent and then, of course, I’m calling because my gums are bleeding.” “Of course, it’s different; it’s because of the priority. It’s not the same, a priority 0 enter the queue, and it’s different.” “The wait is between the telephone operator and the health professional, and there it can be 10 min.”	18	“I think they could have made me wait less time considering that it’s not a free phone call.” “They assist you quickly over the phone, but there is some delay in transferring you to the health professional;” “It depends upon the day, sometimes they don’t take long while other times they are slow;” “I had to wait a long time before they responded.” “A receptionist helps you first, taking your information, and then transfers you to a doctor or nurse.” “After talking to the first person, they transferred me directly to the health professional.”	8
2.3 Repetition of the same information to different professionals			
Request for the same information from the caller/user by different professionals “He takes some information and transfers the call;” “This makes the interrogation very difficult because the caller is already annoyed.” “By time the physician arrives, the patient has repeated the same thing 3 times.” “People complain that they repeat everything many times, that you always ask the same questions;” “This is already the fourth or fifth time!” “I have to say the same thing again?”	5	User/Caller assesses the questions asked as necessary concerns “They asked me the questions necessary for resolving my problem.” “They were very concise with the information they asked me about.” “The questions they ask are those necessary for learning what is happening to me.”	7
2.4 Identification of the professional and his/her role			
Role of the professional is not identified or assessed by the user/caller			
“Sometimes they don’t listen, you know? I introduce myself as a nurse, but… ”“When the person attending is a nurse, it is sometimes necessary to make the user understand the nurse is completely capable of fulfilling her role.” “Many believe that a physician should take care of them.”	8	“I don’t recall them telling me what type of professional he was.” “I think the person who resolved the consultation was a doctor.” “A doctor and a nurse took care of me.” “The person who responds to your consultation is identified by the type of profession, but not by name.”	11
3. Assessment of the care provided over the telephone			
3.1 Professionalism of the personnel who answered the telephone			
The perception that professionalism increases the level of confidence in the user/caller			
“And tell them we are going to help them.” “Speak with him and calm him down.” “Don’t be hesitant; I think he senses that it’s clear to you.” “I believe that what they want, depending upon the type of consultation, is to know what they can find and how they have to act. The uncertainty about how it might go for them causes some anxiety that you can lower if you give them that information.”	15	“I had the feeling that the person who treated me was a good professional.” “You can tell that they know about and control the information that you need.” “It seemed to me that the professional knew what she was doing.”	6
3.2 Comprehension of the information provided by the caller/user			
Elements that make the user/caller perceive that his/her demand is understood			
“I repeat back what she has told me.” “The funny thing is that some times, if you listen until the very end, you do not resolve anything, but they literally say thank you for listening to me.” “Speak at the patient’s level.”	8	“I felt like I was understood.” “He asked me the necessary questions to understand what I needed.” “Most times they understand you, but it also depends upon the problem.”	7
3.3 Kindness and empathy toward the user/caller			
Sensation of kindness and capability of handling the emotions of the user/caller			
“But they call right away to ask for assistance, and then you reassure them?” “Try to empathize with the patients. I understand that it is like this…”	5	“The professional who helped me was very kind.” “She treated me very well.” “They showed an interest in what was happening to me and were very kind to me.”	6
3.4 Clarity of the information			
Perception that the professionals provide clear information			
“The information provided by the professional must be clear.” “Attempt to provide answers to the consultation.”	3	“The information provided by the professional was clear.” “He did not adapt the information to my level and so it was very difficult for me to understand what he was talking about.”	2
3.5 Resolution of the problem			
Resolving the problem that they have called about is essential for assessing the quality of the service			
“The most important thing for assessing the quality of the call is to resolve the problem they called about.” “The emergency service resolved the problem for me.” “Try to provide answers the consultation.”	3	“They resolved the problem I called about.” “They did not answer my consultation.” “They told me I would receive the individual health card (TSI) within two weeks, but months passed.” “The resolved my consultation;” “Most of the times that I have called, they gave me the answer that I needed.”	7

**Table 4:** Subprocess 3: Urgent Healthcare Transport.

Professionals		Caller/User	
4. Mobilization of the resource			
4.1. Arrival time of the healthcare resource			
Arrival time in urban areas between 5 and 10 min after receiving of the call			
“If it is within the coverage area, between 5 and 8 min.”	5	“The truth is that whenever I have called, they have taken 15 min at the most.” “061 gets there right away.”	9
4.2. Telephone accompaniment until the professional assistance arrives			
In most cases, there is no telephone accompaniment except for very specific situations			
“Maintaining contact with the caller to guide the ambulance is not common because in some cases it may take half an hour.” “If the caller is on a ledge and tells you he is going to jump, you send the assistance but keep speaking with him.”	2	“They did not remain on the line until the ambulance arrived.” “As I was by myself, they told me to open the door, and they remained with me until the ambulance arrived.” “In some serious situations, it is necessary for the professional to remain on the line.”	5
5. Arrival of the assistance and care provided in situ			
5.1. Assisting team			
Difficulty of the caller in identifying the professional profile of the assisting personnel			
“The caller does not identify between technicians and physicians.”	3	“I thought two nurses were coming.” “I don’t know what to say. There were two persons looking after me the whole time.”	8
5.2. Care received			
Special attention in transmitting confidence in the decisions made by the personnel		Care received that is perceived as correct and complete	
“We say it confidently and explain why.” “They need to see us calm, and not act like chickens with their heads cut off.”	3	“The look at my oxygen levels, everything.” “They did several tests; they drew blood, and took my blood pressure.”	6
5.3. Information			
Explanations given to the user or family on care given and decisions made			
“We try to explain to the user everything that we do to him.” “How she is, what she has, where we are taking her, what is the diagnosis.” “What we are doing and why.”	3	“They tell my family everything they do to me.”	1
6. Decisions about the transport			
6.1. Choice of health center			
In most cases, the professionals and the user negotiate the destination center			
“We must negotiate more in the basic assisted transport than in the advanced ambulances because it depends upon the area.” “Here in Barcelona, we negotiate a fair amount, a lot … we almost always end up doing what the user demands even though it might not be the most suitable site.”	5	“They decided to take me to the hospital that does everything to me.” “They asked me what I thought about them taking me to my hospital and I said that was fine because it was closest to where I lived.” “I already told them to take me to my hospital.”	8
6.2. Notifying family members of the user about the transport			
Despite the inexistence of a protocol about notifying family members, in the cases in which the user requests such notification, it is attempted		Preference of the user him/herself to notify family members	
“At some point, for humanity’s sake, you can notify family members if the user is elderly and unable to call.” “Sometimes they ask you, ‘Can you please call my daughter?’” “For humanity’s sake, not due to some protocol.”	4	“I wanted to notify my family so that they wouldn’t get any more alarmed than necessary.” “The team of professionals did not notify my family because I refused.” “I kept my family informed with my cell phone.”	3
7. Care during transport			
7.1. Safety during transport			
Safety mechanisms and protocols during vehicular transport for both users and professionals	2	Perception that the vehicle moves quickly but does so safely	1
“The little girl went with her harness and the nurse rode in the cab because everybody has to be seated.” “Safety is paramount. No professional may travel standing up.”		“They go fast but do so normally and safely.”	
7.2. Accompaniment by family members during transport			
Possibility for the user to be accompanied by a family member within the cabin with exceptions for pediatric cases			
“As a driver, you try to provide support for the family member who rides beside you during the transport. You try to redirect and control the situation so that when they reach the hospital, they feel a bit better.” “The mother or father always rides in the back with the child if the child is relatively stable. If we anticipate that the condition may worsen, then the family members ride up front.” “In a critical situation, the parent rides up front in the cab.”	2	“My family member rode up front.” “My mother also went along with me, in the part with the driver.”	3
7.3. Safekeeping of personal belongings			
Despite the inexistence of a protocol for collecting and safeguarding a user’s personal belongings when found in a public location, the personnel offer to take such belongings with them to the center of destination.		On some occasions, the personnel at the receiving center offer to safeguard a user’s personal belongings whereas on others, the user decides to take them with him/herself	
“We try to give the user’s belongings to the police, but many times, they do not want them.” “Part of the report that we deliver in the hospital includes the user’s belongings. If they are valuable, they are turned over to a user’s family member, and the hospital is notified.”	2	“If you are in the ER and lucky, a professional appears, they safeguard your belongings.” “As I had my backpack with me, wherever the stretcher goes, so goes the backpack. I prefer not to leave my things in the hands of others.”	2
8. Arrival at the health center			
8.1. Reception by the personnel at the receiving center			
Triage is the moment in which the emergency healthcare transport personnel transfer responsibility for the user to the personnel at the receiving center			
“Upon arrival at the center, triage is performed; at that moment, the user realizes that he is being taken care of by hospital personnel, and we move him from the stretcher to a hospital bed. It is at that moment that we disappear.” “The transport personnel inform the center personnel that they are passing the user to them, and at that moment, we leave the user in their care. When the user is removed from the stretcher, the activity by the transport personnel ceases.”	2	“Upon arrival at the center, we wait in the ER, they take you to triage, and you wait until your turn.”	1
		Perception that an effective transfer of information is not always accomplished by the transport personnel to the personnel at the receiving center	
		“Hospital is completely full; they do not find anyone to leave me with, so many times, the transfer of information remains in the air.” “The transport personnel should insist a little bit in finding a doctor, because they leave you there with the information that you gave them.” “It depends upon the occasion. Sometimes, they leave you there with a long wait.”	3

## Supplementary Materials

The following are available online at http://www.mdpi.com/1660-4601/15/2/233/s1, Table S1: Survey Instrument. Caller/User, Table S2: Survey Instrument. Professionals.

## References

[1] Koos E. (1954). The Health of Regionsville.

[2] Donabedian A. (1966). Evaluating the quality of medical care. Milbank Q..

[3] Doll R. (1974). Surveillance and monitoring. Int. J. Epidemiol..

[4] Linder-Pelz S. (1982). Social psychological determinants of patient satisfaction: A test of five hypotheses. Soc. Sci. Med..

[5] Sitzia J. (1999). How valid and reliable are patient satisfaction data? An analysis of 195 studies. Int. J. Qual. Health Care.

[6] Mira J.J. (2006). La satisfacción del paciente: Teorías, medidas y resultados. Todo Hosp..

[7] Hall J., Dornan M. (1988). What patients like about their medical care and how often they are asked: A meta-analysis of the satisfaction literature. Soc. Sci. Med..

[8] Servicios de Urgencias y Emergencias 112/061. Datos 2015. http://www.msssi.gob.es/estadEstudios/estadisticas/estadisticas/estMinisterio/SIAP/Estadisticas.htm.

[9] Hadsund J., Riiskjær E., Riddervold I.S., Christensen E.F. (2013). Positive patients’ attitudes to pre-hospital care. Dan. Med. J..

[10] Swain A.H., Al-Salami M., Hoyle S.R., Larsen P.D. (2012). Patient satisfaction and outcome using emergency care practitioners in New Zealand. Emerg. Med. Australas..

[11] Anisah A., Chew K.S., Mohd Shaharuddin Shah C.H., Nik Hisamuddin N.A. (2008). Patients’ perception of the ambulance services at Hospital Universiti Sains Malaysia. Singap. Med. J..

[12] Mason S., Knowles E., Colwell B., Dixon S., Wardrope J., Gorringe R., Snooks H., Perrin J., Nicholl J. (2007). Effectiveness of paramedic practitioners in attending 999 calls from elderly people in the community: Cluster randomised controlled trial. BMJ.

[13] Bernard A.W., Lindsell C.J., Handel D.A., Collett L., Gallo P., Kaiser K.D., Locasto D. (2007). Postal survey methodology to assess patient satisfaction in a suburban emergency medical services system: An observational study. BMC Emerg. Med..

[14] O’Meara P. (2003). Ambulance satisfaction surveys: Their utility in policy development, system change and professional practice. JEPHC.

[15] Persee D.E., Key C.B., Baldwin J.B. (2002). The effect of a quality improvement feedback loop on paramedic-initiated nontransport of elderly patients. Prehosp. Emerg. Care.

[16] Neumayr A., Gnirke A., Schaeuble J.C., Ganter M.T., Sparr H., Zoll A., Schinnerl A., Nuebling M., Heidegger T., Baubin M. (2016). Patient satisfaction in out-of-hospital emergency care: A multicenter survey. Eur. J. Emerg. Med..

[17] Turner J., Coster J., Chambers D., Cantrell A., Phung V.-H., Knowles E., Bradbury D., Goyder E. (2015). What Evidence Is There on the Effectiveness of Different Models of Delivering Urgent care? A Rapid Review.

[18] Booker M.J., Shaw A.R.G., Purdy S. (2015). Why do patients with ‘primary care sensitive’ problems access ambulance services? A systematic mapping review of the literature. BMJ Open.

[19] Hamam A.F., Bagis M.H., AlJohani K., Tashkandi A.H. (2015). Public awareness of the EMS system in Western Saudi Arabia: Identifying the weakest link. Int. J. Emerg. Med..

[20] Togher F.J., O’Cathain A., Phung V.H., Turner J., Siriwardena A.N. (2015). Reassurance as a key outcome valued by emergency ambulance service users: A qualitative interview study. Health Expect..

[21] Aronsson K., Björkdahl I., Wireklint Sundström B. (2014). Pre-hospital emergency care for patients with suspected hip fractures after falling—Older patients’ experiences. J. Clin. Nurs..

[22] Kietzmann D., Wiehn S., Kehl D., Knuth D., Schmidt S. (2016). Migration background and overall satisfaction with pre-hospital emergency care. Appl. Nurs. Res..

[23] Keene T., Davis M., Brook C. (2015). Characteristics and outcomes of patients assessed by paramedics and not transported to hospital: A pilot study. Australas. J. Paramed..

[24] Studnek J.R., Fernandez A.R., Vandeventer S., Davis S., Garvey L. (2013). The association between patients’ perception of their overall quality of care and their perception of pain management in the pre-hospital setting. Prehosp. Emerg. Care.

[25] Togher F.J., Davy Z., Siriwardena A.N. (2013). Patients’ and ambulance service clinicians’ experiences of pre-hospital care for acute myocardial infarction and stroke: A qualitative study. Emerg. Med. J..

[26] Johansson A., Ekwall A., Wihlborg J. (2011). Patient satisfaction with ambulance care services: Survey from two districts in Southern Sweden. Int. Emerg. Nurs..

[27] O’Cathain A., Knowles E., Turner J., Nicholl J. (2014). Acceptability of NHS 111 the telephone service for urgent health care: Cross sectional postal survey of users’ views. Fam. Pract..

[28] Carrasqueiro S., Oliveira M., Encarnação P. (2011). Evaluation of telephone triage and advice services: A systematic review on methods, metrics and results. Stud. Health Technol. Inform..

[29] Ström M., Marklund B., Hildingh C. (2009). Callers’ perceptions of receiving advice via a medical care help line. Scand. J. Caring Sci..

[30] Soriano C., Soriano F., Morant F. (2011). Análisis de la calidad percibida por los usuarios externos de la Unidad de Coordinación de Transporte Sanitario no Asistido de Alicante. Rev. Calid. Asist..

[31] Beaulieu R., Humphreys J. (2008). Evaluation of a telephone advice nurse in a nursing Managed pediatric community clinic. J. Pediatr. Health Care.

[32] Jones R., Avies-Jones A. (2007). An audit of the NICE self-harm guidelines at a local accident and emergency department in North Wales. Accid. Emerg. Nurs..

[33] Mason S., O’Keeffe C., Coleman P., Edlin R., Nicholl J. (2007). Effectiveness of emergency care practitioners working within existing emergency service models of care. Emerg. Med. J..

[34] Halter M., Marlow T., Tye C., Ellison G.T. (2006). Patients’ experiences of care provided by emergency care practitioners and traditional ambulance practitioners: A survey from the London Ambulance Service. Emerg. Med. J..

[35] Forslund K., Kihlgren M., Ostman I., Sørlie V. (2005). Patients with acute chest pain—Experiences of emergency calls and pre-hospital care. J. Telemed. Telecare.

[36] Mason S., O’Keeffe C., Coleman P., Edlin R., Nicholl J. (2005). A National Evaluation of the Clinical and Cost Effectiveness of Emergency Care Practitioners.

[37] Machen I., Dickinson A., Williams J., Widiatmoko D., Kendall S. (2007). Nurses and paramedics in partnership: Perceptions of a new response to low-priority ambulance calls. Accid. Emerg. Nurs..

[38] Snooks H., Kearsley N., Dale J., Halter M., Redhead J., Cheung W.Y. (2004). Towards primary care for non-serious 999 callers: Results of a controlled study of “Treat and Refer” protocols for ambulance crews. Qual. Saf. Health Care.

[39] Cariello F.P. (2003). Computerized Telephone Nurse Triage. An Evaluation of Service Quality and Cost. J. Ambul. Care Manag..

[40] O’Cathain A., Turner J., Nicholl J.P. (2002). The acceptability of an emergency medical dispatch system to people who call 999 to request an ambulance. Emerg. Med. J..

[41] O’Cathain A., Munro J.F., Nicholl J.P., Knowles E. (2000). How helpful is NHS Direct? Postal survey of callers. BMJ.

[42] Quintana J.M., González N., Bilbao A., Aizpuru F., Escobar A., Esteban C., San-Sebastián J.A., de-la-Sierra E., Thompson A. (2006). Predictors of patient satisfaction with hospital health care. BMC Health Serv. Res..

[43] Mira J.J., Tomás O., Pérez-Jover V., Nebot C., Rodríguez-Marin J. (2009). Predictors of patient satisfaction in surgery. Surgery.

[44] Petek D., Kersnik J., Szecsenyl J., Wensing M. (2011). Patients’ evaluations of European general practice—Revisited after 11 years. Int. J. Qual. Health Care.

[45] Bjertnaes O.A., Strømseng Sjetne I., Iversen H. (2012). Overall patient satisfaction with hospitals: Effects of patient-reported experiences and fulfilment of expectations. BMJ Qual. Saf..

