# Supplementary material for: Patient Satisfaction with Pre-Hospital Emergency Services. A Qualitative Study Comparing Professionals’ and Patients’ Views
Source: Int J Environ Res Public Health. 2018 Jan 30;15(2):233. doi: 10.3390/ijerph15020233 (PMC5858302; doi:10.3390/ijerph15020233)
Supplement: Supplementary file 1 [file ijerph-15-00233-s001.pdf]

Appendix 1. Survey Instrument. Caller/User

**Caller/User**
**112/061 ACCESS**
- First of all, do you remember what telephone number you called for the information consultation?
- Why did you decide to call that number?
- Did they answer the call quickly or did you have to wait?
- Did you have to explain your problem to various persons?
**ATTENTION PROVIDED BY THE DISPATCHER**
- Once a professional assisted you, would you be able to identify whether such professional was a dispatcher, physician, or nurse?
- Did the person indicate his name and professional profile?
- Do you consider that the professional who assisted you understood the need for the information that you were requesting?
- Would you say that they were friendly with you during your consultation?
- How long did the telephone call last?
- Was the information they gave you clear?
- Did they resolve the problem or the consultation you were calling about?
- Were you satisfied with the information that they gave you?
- Did you have to call back another time for the same consultation that day?
**CARE (TELEPHONE ADVICE)**
- From the time you spoke to the dispatcher until you were transferred to a physician or nurse, did you wait a long time?
- Did the attending professional identify herself? Did that person say if she was a physician or nurse?
- Did they ask you many questions?
- Do you think you were assisted by a good professional?
- Did the professional properly understand the questions you were asking, and did she resolve them?
- Did the information that you provided help to resolve your problem?
- Would you say that you were treated with kindness?
- Did you feel that they dedicated all the time that was necessary to resolve your consultation?
- How long did the telephone call last?
- Did the professional who assisted you contact you again to see if your problem had been resolved? (This question is based on the type of consultation that was made, for example, if the consultation was for a child's fever to see if there was a subsequent phone call to inquire whether the fever went down).
- Did you have to call back another time for the same consultation that day?
**MOBILIZATION OF THE RESOUCE. WAIT TIME**
- Until the ambulance arrived, how much time passed-did it arrive quickly or slowly?

Caller/User
- Did they remain on the telephone with you until the ambulance arrived?
- Did they call you at any moment while you waited to confirm whether or not the ambulance had yet to arrive?
**ARRIVAL OF THE RESOURCE. TYPE OF VEHICLE. CARE PROVIDED AT HOME/PUBLIC PLACE**
- How many vehicles assisted you?
- Who took care of you? Were you able to identify whether they were physicians/nurses/technicians?
- Did the professionals act quickly at all times? Did they know how to take care of you? Did they have the proper material with them?
- Did they provide you with any information while they looked after you?
- Were any family members with you? Did they inform such family member/s about what was happening to you?
**ARRIVAL OF THE RESOURCE. Decision about the transport**
- How is the decision to transport the patient to the center made?
- Did they notify any family member of yours that they were taking you to a center?
- When they transported you, did they tell you that we're going to such and such a center because its closest, or did they ask you if you had any preferences on which centers to go to?
**MOBILIZATION OF THE VEHICLE-TRANSPORT. Care during the transport**
- How was the transport? Did you feel comfortable, secure, at ease? Did anybody accompany you in the ambulance? Was it noisy?
- Did they tell you anything while on the way to the center? Something like, "Do not worry, were are on our way, they are waiting to help you." Do you think the treatment you received while inside the vehicle was adequate?
- Did you have any personal items with you? What happened to them? Who safeguarded them?
**ARRIVAL AT THE CENTER**
- What happened when you arrived at the center?
- Were they waiting for you?
- Did the ambulance crew say goodbye to you?
- Did they tell you what was going to happen to you from that time forward?
- If you had to use this service again, would you improve anything about it? What was positive about it?

Appendix 2. Survey Instrument. Professionals

Professionals
**Key elements in the Catalonia Medical Emergency Service process**
- How is the demand received and classified?

**Professionals**
- When is the demand resolved directly by the dispatcher?
- When is a call passed to the second level and is any telephone advice offered?
**ATTENTION PROVIDED BY THE DISPATCHER**
- According to your opinion, how many calls are there on hold in your environment on a normal day while others are being attended to?
- In your opinion, in which cases is the user not satisfied with the response?
- In how many cases do you call the user back for any reason to reconnect?
- What types of cases do not receive an adequate response?
- What do you do on a regular basis so that the user understands your question or request?
- Are you confident about your responses?
- Do they perceive agility in the response given to them?
**CARE (TELEPHONE ADVICE)**
- How do you manage to get the user to feel that his/her petition is understood?
- How do you get users to trust the questions they are asked and the responses they are given?
- Is your professionalism perceived?
- How do you teach them, how do you empower the user for similar situations that may occur in the future?
- How do you make the user feel a sense of agility in the response?
- How do you make the user perceive that you know how to handle the emotions that he feels?
- How do you transmit calm and safety about what must be done with your voice and while following the appropriate protocol?
- How do you manage to make the proposed solution satisfy the user's expectations?
- In cases of emergency, do you remain in contact with the patient, or do you make contact again after a period, for example until the ambulance arrives?
**MOBILIZATION OF THE RESOURCE**
- When does the urgent healthcare transport action begin?
- Who are usually the callers?
- What information is needed from the caller and how is the action take out decided upon?
**ARRIVAL OF THE RESOURCE. TYPE OF VEHICLE. CARE AT HOME/PUBLIC PLACE**
- How do they find the destination?
- What resources are activated depending upon the cases, and how are the resources sent?
- How many vehicles are mobilized?
- Until they arrive at the destination, does any type of action take place?
**ARRIVAL OF THE RESOURCE. Decision on the transport**
- Are the types of attending professionals (physician, nurse, technician) identified?
- What information do the professionals provide?

**Professionals**
- If the patient is transported, how does this go about? How is the patient told? How are family members told?
- In the case of a multiple accident, how do they organize themselves to attend to the patients?
- In the case of a family accident, how is the transport for each family member decided upon?
- How do they make the user confident in the decisions that they make?
- How do they make the user perceive that they are good professionals?
- How do they make the user perceive that they know how to manage emotions?
- How do they make the user perceive that the responses are agile?
**MOBILIZATION OF THE VEHICLE-TRANSPORT. Care during the transport**
- Is any action performed on the patient during the transport?
- Is the patient given any information?
- What happens to patients' personal items? Does any protocol exist for them?
- What do they do to transmit a sense of security and confidence during the time that they are carrying out the transporting service?
**ARRIVAL AT THE CENTER**
- How do they make the transport to the health centers or hospitals appropriate?
- How is the arrival of the emergency services organized?
- Did the ambulance crew say goodbye to you?
